# Supplementary figures and images for: Adaptive evolution of Moniliophthora PR-1 proteins towards its pathogenic lifestyle
Source: BMC Ecol Evol. 2021 May 14;21:84. doi: 10.1186/s12862-021-01818-5 (PMC8120714; doi:10.1186/s12862-021-01818-5)

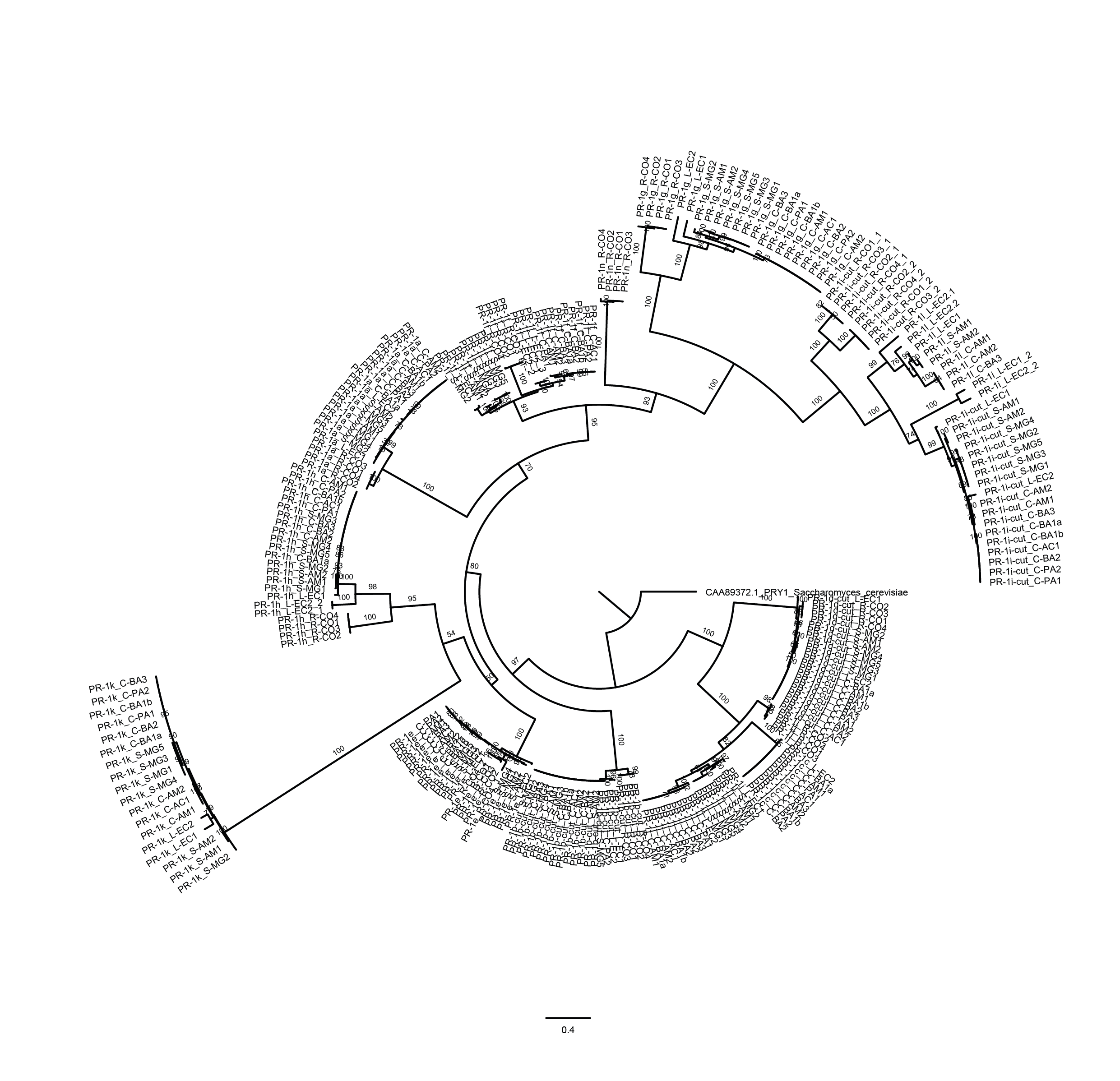

Supplement: Supplementary file 4 — Additional file 4. Phylogenetic reconstruction of PR-1 proteins in Moniliophthora isolates (version with non-collapsed branches). Figure of phylogenetic tree with non-collapsed branches of PR-1 proteins identified from genomes of 18 M. perniciosa and 4 M. roreri isolates, inferred by maximum likelihood and branch support obtained using 1000 bootstraps. The PRY1 protein of Saccharomyces cerevisiae was used as an outgroup. [file 12862_2021_1818_MOESM4_ESM.png]

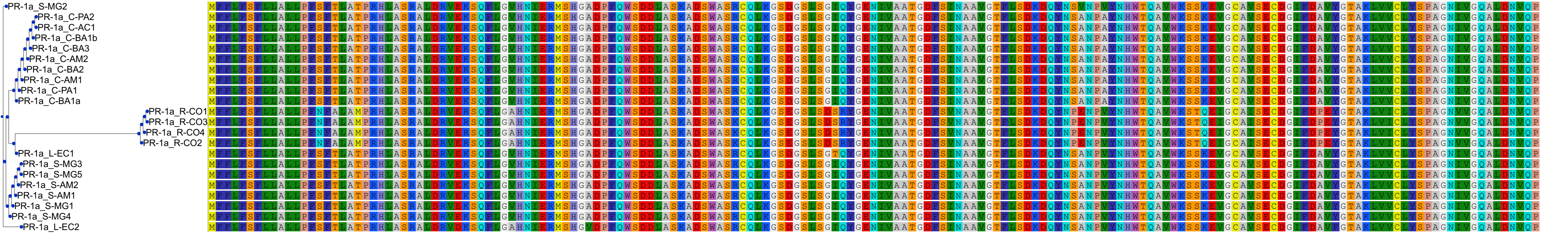

Omega value for sites under M2.0.2\_PR-1a model

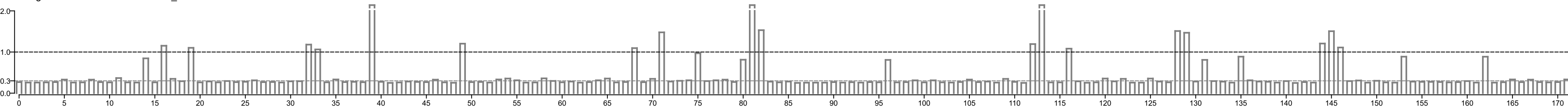

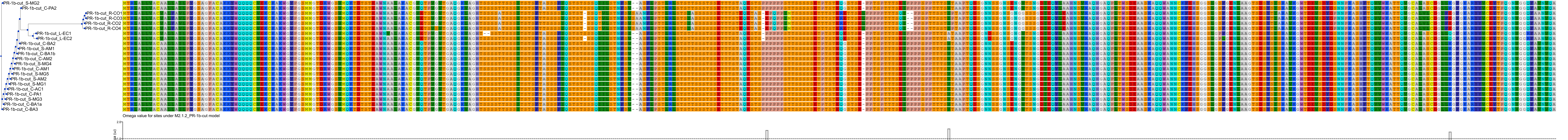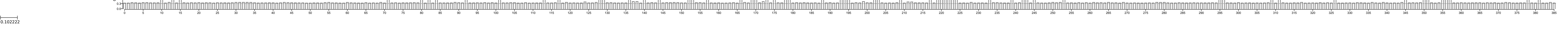



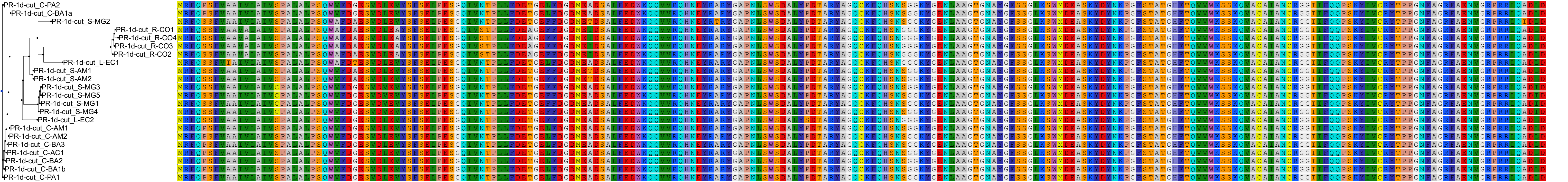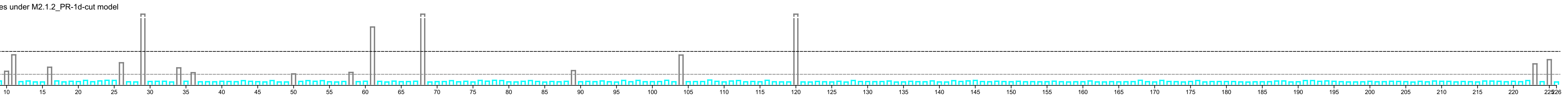

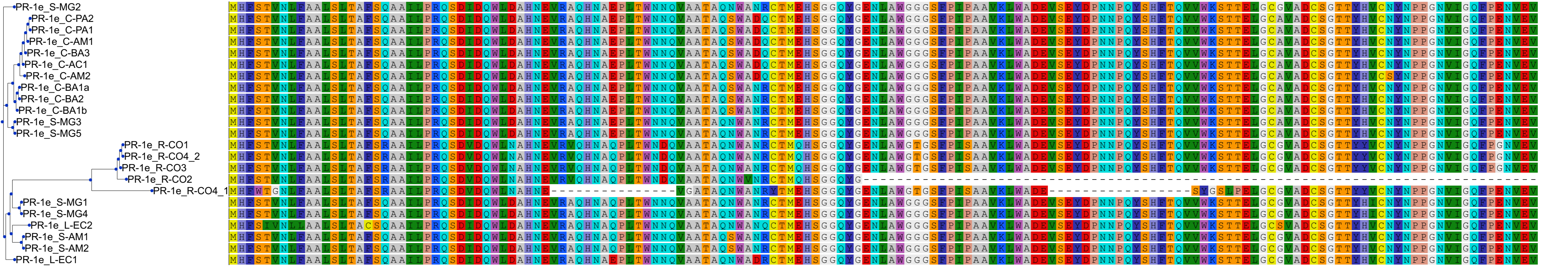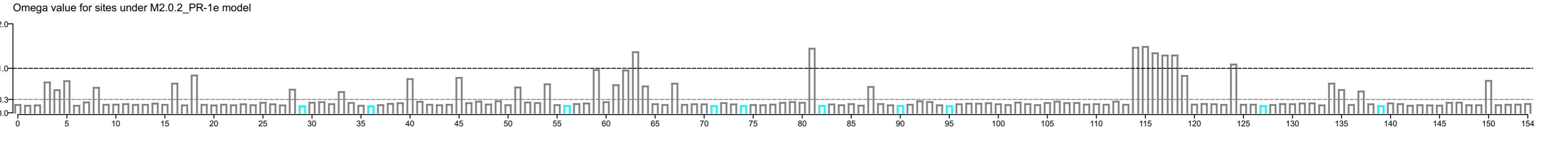

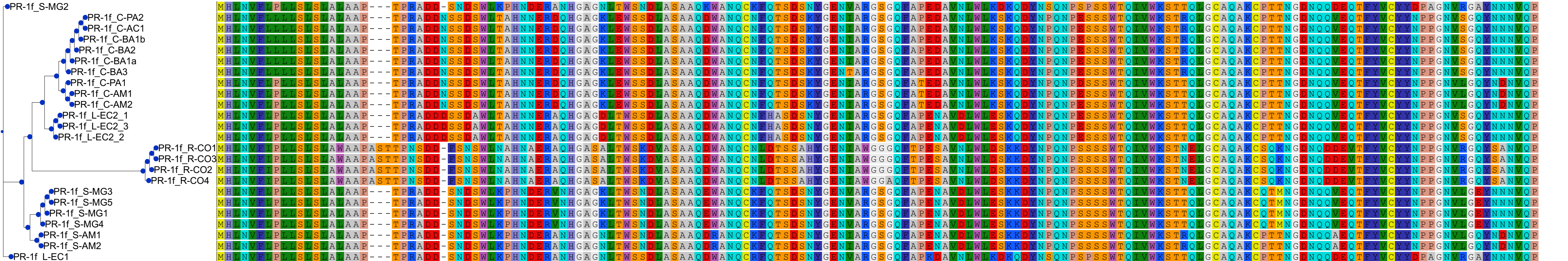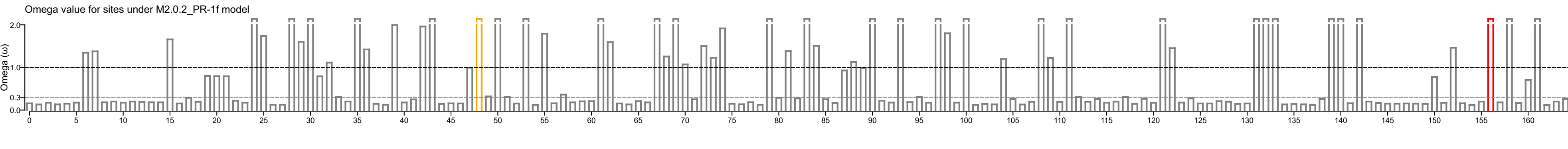

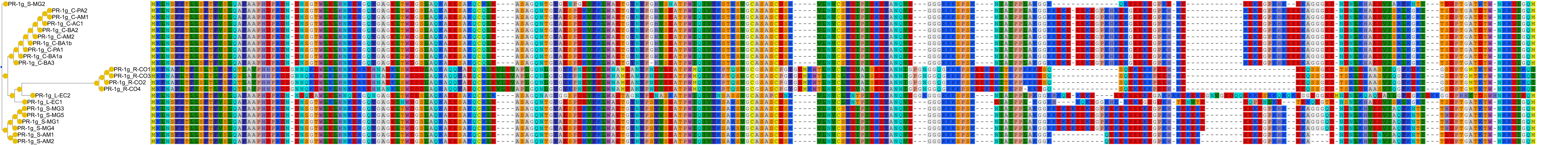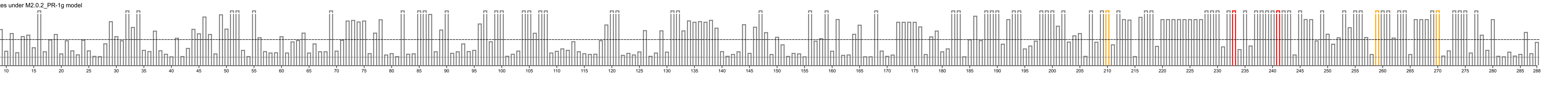

0.080611

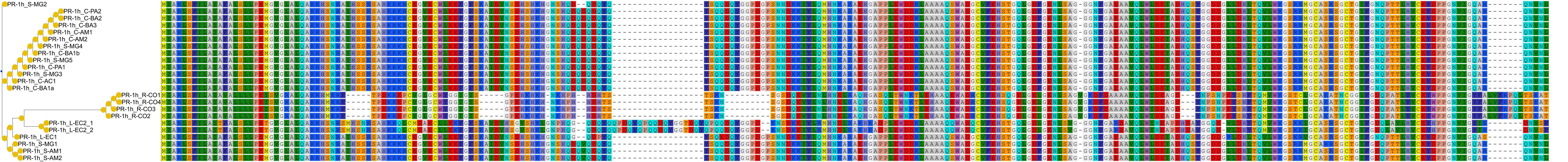

0.68

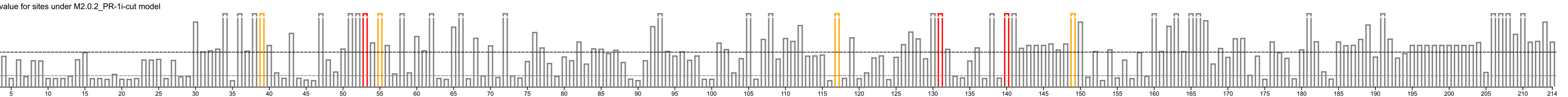



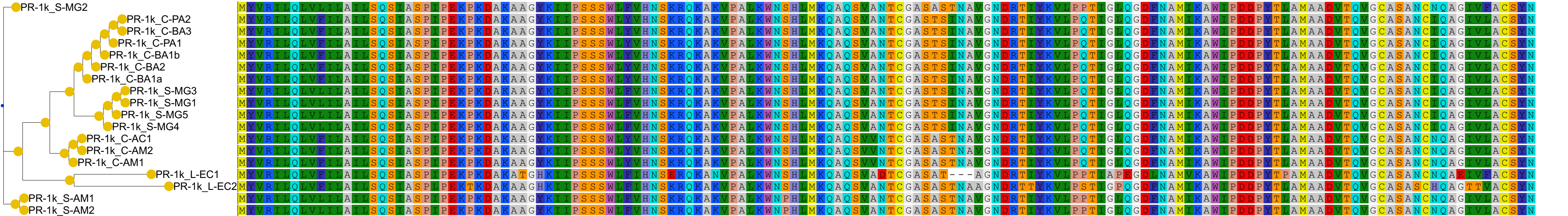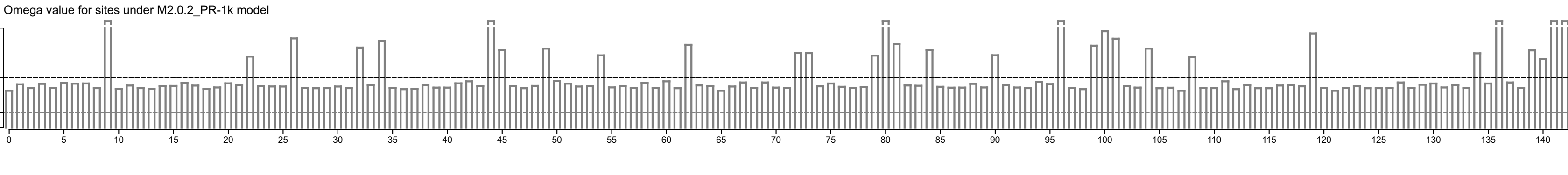

Supplement: Supplementary file 5 — Additional file 5. Protein sequence alignment and omega (dN/dS) values of each PR-1i family of Moniliophthora genomes. PDF file containing figures of the protein sequence alignment for each PR-1 family from Moniliophthora isolates and a bar chart of omega (dN/dS) values calculated for each amino acid site along the alignment using the site model test of codeML. Sites with positive selection signs are indicated by red (p-value ≤ 0.01) or orange (p-value ≤ 0.05) bars in the bar chart of omega values below the alignment. Figures are organized by alphabetical order of the names of PR-1 families. [file 12862_2021_1818_MOESM5_ESM.pdf]
